# Between the scalpel and algorithms: how AI is redesigning Surgery

**DOI:** 10.1590/0100-6991e-20250031EDIT01-en

**Published:** 2025-12-23

**Authors:** CRISTIANO XAVIER LIMA, MARCOS ANDRÉ GONÇALVES, ARTHUR DE OLIVEIRA LIMA

**Affiliations:** 1 - Universidade Federal de Minas Gerais (Departamento de Cirurgia) - Belo Horizonte - MG - Brasil; 2 - Universidade Federal de Minas Gerais (Departamento de Ciências da Computação) - Belo Horizonte - MG - Brasil; 3 - Politecnico di Torino (Dipartimento di Automática e Informatica) - Turim - Itália

## INTRODUCTION

Surgery has always been at the confluence of science, technology, and human skills. In recent decades, the field has expanded far beyond the operating room. Perioperative medicine, multimodal cancer treatment, robotic platforms, and complex transplants are now part of everyday reality^1^. This rapid progress brings undeniable benefits, but it also generates a paradox: surgeons are expected to master an ever-growing body of knowledge, while providing safe and timely care in more challenging situations^2^. The result is a widening gap between the pace of innovation and the ability of individuals and health systems to absorb it. At the same time, the surgical workforce faces increasing demands, from training new generations in advanced techniques to dealing with the ethical and economic implications of recent technologies^3^. In this scenario, the challenge is no longer access to information, but the ability to transform enormous amounts of data into clinically meaningful decisions. Artificial intelligence, and particularly large language models (LLMs), have emerged as disruptive tools, with the potential to reshape the way surgeons learn, teach, and practice^4^.

Natural language processing (NLP) has long been explored in medicine, from extracting structured information from electronic health records to supporting clinical documentation and mining literature^5^ ^-^ ^6^. These earlier applications, while valuable, were limited to constrained and predefined tasks. The recent emergence of LLMs represents a qualitative leap in this field, moving from rules-based systems to versatile models, capable of understanding context, generating coherent narratives, and participating in interactive dialogues^7^ ^-^ ^8^. For the field of surgery, this evolution is not merely incremental: LLMs bring the possibility of transforming the way surgeons learn complex procedures, teach future generations, and integrate evidence into daily practice^9^.

The roots of LLMs trace back to advances in machine learning, particularly deep learning and transformative architectures introduced in 2017, which enabled the handling of long strings of text with unprecedented efficiency^8^. Unlike traditional statistical or rule-based NLP systems, LLMs are pre-trained on vast sets of heterogeneous texts and then adapted for domain-specific tasks, allowing them to generalize across different contexts^7^. State-of-the-art models - such as GPT-4^10^ and instruction-adjusted variants such as Flan-PaLM^11^ - encode not only linguistic structures but also factual and procedural knowledge. Its versatility lies in its ability to process unstructured data, generate summaries, translate languages, and simulate reasoning patterns^12^. In the clinical domain, these capabilities translate into more agile access to knowledge, real-time educational support, and improved communication between multidisciplinary teams^9^. For surgeons, advantages include the ability to quickly retrieve best practices, draft structured operative reports, support decision-making with synthesized evidence, and provide adaptable teaching resources that align with the student’s level of expertise^4^.

The integration of LLMs into medicine follows a timeline that reflects both technological readiness and clinical adoption. Early milestones in general medicine include the use of NLP for automated coding in the 1990s^5^, the first clinical decision support systems in the 2000s^13^, and the success of instructional-adjusted LLMs, such as Flan-PaLM^11^, in improving performance in medical benchmarks after their introduction in the early 2020s^14^. Since 2022, scientific papers have demonstrated that models such as Flan-PaLM achieve cutting-edge accuracy in MultiMedQA tasks, including an accuracy of over 67% in USMLE^14^ type questions. In surgery, adoption lagged slightly behind, but it is accelerating rapidly: initial explorations around 2021 focused on automating operative notes^15^; in 2022, feasibility studies demonstrated the usefulness of LLMs in generating perioperative checklists^16^; and as of 2023, the first educational platforms have started to integrate LLMs into simulation and surgical training modules^17^. This dual trajectory underscores a progressive shift: from administrative support in general medicine to clinically relevant, procedure-oriented applications in surgery, with each stage requiring rigorous validation before integration into practice.

LLMs are now at the forefront of medical AI and have exciting potential in clinical work, education, and research^9^. The barriers to immediate implementation in these three domains represent opportunities for further development that can be exploited by LLM developers and independent research teams. Currently, the use of LLMs is still limited in medicine, due to their lack of precision, timeliness, coherence, and transparency, as well as for ethical reasons^18^ ^-^ ^4^. LLM technology can, however, have a substantial impact on the way medical work is conducted, particularly where risks are lower, personal data is not needed, and specialist knowledge is not required or provided by the user^19^.

In recent years, several emerging applications of large language models have been identified within the field of surgery^15^ ^-^ ^17^. Looking ahead, one of the most promising proposals is the integration of LLMs with vision-language models (VLMs). While VLMs can interpret surgical images and videos, they often lack the ability to contextualize their results within clinical workflows. LLMs can enhance these systems by translating complex visual patterns into clinically meaningful narratives, linking intraoperative findings to evidence-based knowledge, and generating feedback for surgical training. This multimodal integration could transform perioperative decision-making and education, but it also amplifies the need for rigorous validation to avoid biases or oversimplified standardizations.

Some proposals for VLMs have recently been presented specifically for the surgical context, including prototypes such as SurgicalGPT^20^, Surgical-VQA^21^, Surgical-VQLA^22^ and Surgical-LVLM^23^. These systems combine the extraction of visual features with LLMs to interpret surgical scenes, classify procedural phases, or answer structured questions about tools and steps. While these approaches demonstrate the feasibility of multimodal AI in surgical settings, they remain experimental. Most models focus on narrow classification tasks rather than producing clinically useful narratives. Training is restricted to a few surgical datasets, and transparency is limited, with only one open-source model available for independent validation. In contrast, medical VLMs, such as Med-Gemini and GPT-4 Omni, are closed-source and do not clearly disclose the extent of surgical data in their training. This picture illustrates both the promise and immaturity of VLMs in surgery: they highlight the next frontier of multimodal integration, but also reinforce the need for rigorous validation, transparency, and surgical leadership before these tools can safely enter clinical practice.

Nevertheless, the field still lacks rigorous and pragmatic trials capable of validating these tools in real surgical environments. If surgeons do not take the lead in developing and validating LLM-based applications, there is a concrete risk that such technologies will be designed without accounting for the unique complexities of surgical care. These systems may introduce biases, promote unsafe standardizations, and overlook crucial intraoperative variables that cannot be captured in text-based datasets.

Beyond the operating room, the absence of surgical leadership may distort the use of these technologies in medical education-either by oversimplifying procedural steps or by providing inadequate guidance to trainees-and ultimately jeopardizing both patient safety and professional development.

Given that surgery has historically embraced innovation-from anesthesia to minimally invasive techniques and robotics-the surgical community now faces a new responsibility: to critically test, refine, and guide the integration of LLMs into practice, education, and research. If surgery is to remain at the forefront of medical innovation, surgeons must not only adopt LLMs but also lead their rigorous validation and ethical incorporation into clinical practice.
